# Fungal Biofilm: An Overview of the Latest Nano-Strategies

**DOI:** 10.3390/antibiotics14070718

**Published:** 2025-07-17

**Authors:** Andrea Giammarino, Laura Verdolini, Giovanna Simonetti, Letizia Angiolella

**Affiliations:** 1 Department of Public Health and Infectious Diseases, Sapienza University of Rome, Piazzale Aldo Moro 5, 00100 Rome, Italy; 2 Department of Environmental Biology, Sapienza University of Rome, Piazzale Aldo Moro 5, 00185 Rome, Italy; giovanna.simonetti@uniroma1.it

**Keywords:** biofilm, nanoparticles, *Candida* spp., *Aspergillus* spp., antimicrobial resistance, antifungal activity

## Abstract

**Background/Objectives**: There is an increasing incidence of fungal infections in conjunction with the rise in resistance to medical treatment. Antimicrobial resistance is frequently associated with virulence factors such as adherence and the capacity of biofilm formation, which facilitates the evasion of the host immune response and resistance to drug action. Novel therapeutic strategies have been developed to overcome antimicrobial resistance, including the use of different type of nanomaterials: metallic (Au, Ag, Fe_3_O_4_ and ZnO), organic (e.g., chitosan, liposomes and lactic acid) or carbon-based (e.g., quantum dots, nanotubes and graphene) materials. The objective of this study was to evaluate the action of nanoparticles of different synthesis and with different coatings on fungi of medical interest. **Methods**: Literature research was conducted using PubMed and Google Scholar databases, and the following terms were employed in articles published up to June 2025: ‘nanoparticles’ in combination with ‘fungal biofilm’, ‘*Candida* biofilm’, ‘*Aspergillus* biofilm’, ‘*Cryptococcus* biofilm’, ‘*Fusarium* biofilm’ and ‘dermatophytes biofilm’. **Results**: The utilization of nanoparticles was found to exert a substantial impact on the reduction in fungal biofilm, despite the presence of substantial variability in minimum inhibitory concentration (MIC) values attributable to variations in nanoparticle type and the presence of capping agents. It was observed that the MIC values were lower for metallic nanoparticles, particularly silver, and for those synthesized with polylactic acid compared to the others. **Conclusions**: Despite the limited availability of data concerning the stability and biocompatibility of nanoparticles employed in the treatment of fungal biofilms, it can be posited that these results constitute a significant initial step.

## 1. Introduction

Nowadays, the number and severity of fungal infections are steadily increasing, causing more than 6.55 million fungal infections per year with an attributable mortality rate of about 2.5 million [1]. The increase in the number of infections is accompanied by the resistance of these microorganisms to available drug therapies. Patients with compromised immune systems due to diseases or drug therapies, such as HIV, chemotherapy and immunotherapy for cancer, organ transplants and premature infants, are most at risk of infection. It is important to note that the pandemic of the novel coronavirus, designated as SARS-CoV-2, has been associated with an increased incidence of concomitant fungal infections [2,3,4]. The lack of new therapeutic classes being approved combined with the increase in incidence led to the publication of a list of priority fungal pathogens (FPPL) by the WHO, with the aim of directing scientific research towards the study of these microorganisms and possible therapeutic alternatives to overcome drug resistance [5]. Biofilm formation plays a big role in the instauration of drug resistance. A biofilm is a community of microorganisms that, after adhering to a surface, biotic or abiotic, starts producing an extracellular matrix (ECM) from polymeric substances to embed and protect its cells. Fungal cells in biofilms are up to 2000 times more resistant to antifungal agents, compared to cells in planktonic form, as the result of physical barriers, altered gene expression and reduced metabolic activity (pmc.ncbi.nlm.nih.gov). The ECM and the biofilm structure protect fungal cells from host immune defenses, allowing the infection to persist [6]. The biofilm enables the microorganism to survive in hostile environments like abiotic materials, which poses a significant threat to hospitalized patients with compromised immune systems, often due to drug therapies or concomitant infections [7].

The effective treatment of fungal biofilm infections often requires high-dose antifungal therapy up to the surgical removal of the infected device: this increases the risk of side effects and healthcare costs [8]. These are the motives for research into the study of new treatment strategies. The new frontiers comprehend specific antifungal agents, isavuconazole and ibrexafungerp, synergistic combination therapy and nanoparticles [9]. This review focuses on the effect of nanoparticles against biofilms formed by human pathogenic fungi, specifically including *Candida* spp., *Aspergillus* spp. and other clinically relevant genera. The aim is to evaluate recent advancements in this promising delivery strategy and its advantages over traditional antifungal treatments.

## 2. Fungal Biofilm

A biofilm is defined as a community of microorganisms that are irreversibly attached to a given surface, inert material, or living tissue. These microorganisms are known to produce extracellular polymers, which in turn provide a structural matrix for the biofilm [10]. Mature biofilms can release cells in the surrounding medium in a process that is referred to as dispersal [11,12]. The biofilm of fungal pathogens is frequently associated with elevated resistance to pharmaceutical treatments, as it facilitates the evasion of the action of administered drugs and the action of the host’s immune system [13,14].

### 2.1. Candida *spp.*

**Figure 1:**
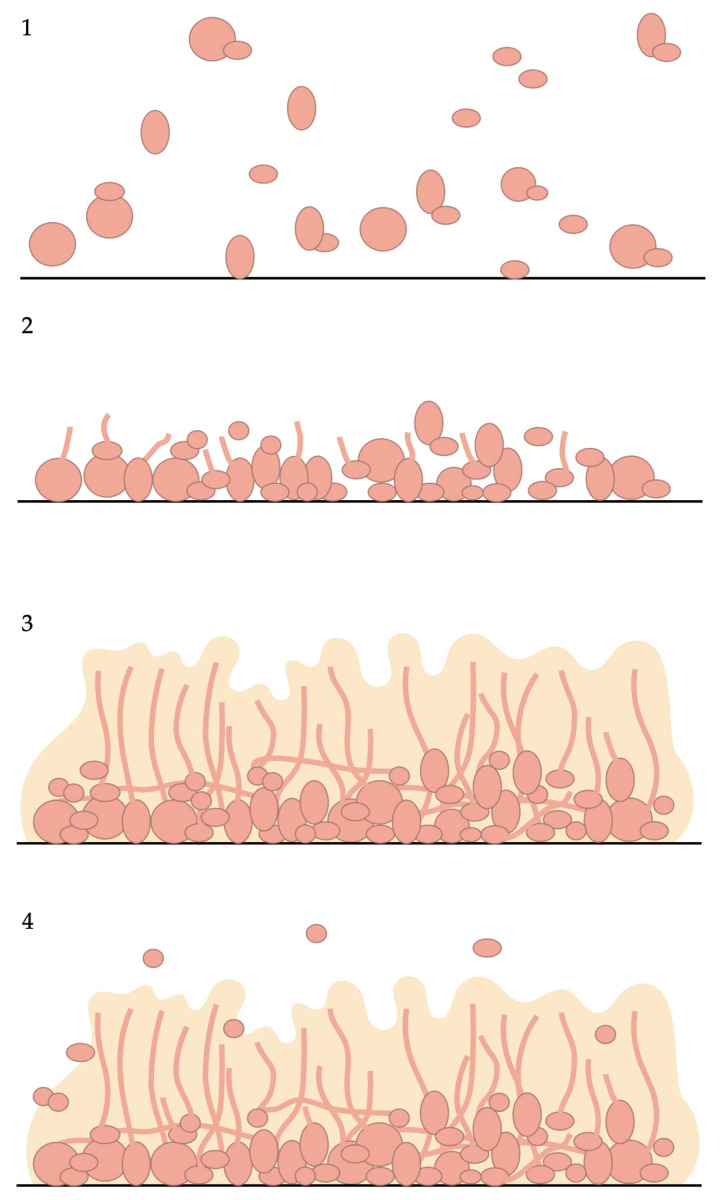
Steps of biofilm formation in *Candida* spp. (**1**) adhesion; (**2**) proliferation; (**3**) maturation; (**4**) dispersion.

During the last decade, *Candida* species have been reported as the most frequent cause of fungal infections in hospitalized and immunocompromised patients. Candidemia is the most common manifestation of invasive candidiasis and is caused by superficial colonizing isolates, including catheter tip or gastrointestinal tract isolates [15]. Currently, more than 150 species of *Candida* have been described, and at least 17 out of these are pathogenic [16]; however, 90% of invasive candidiasis (IC) are caused by *C. albicans*, *C. glabrata*, *C. krusei*, *C. parapsilosis*, *C. tropicalis* [17] and *C. auris*, an emergent virulent and multi-drug-resistant yeast [18]. Among virulence factors, biofilm formation is of special interest because these are highly organized structures containing sessile cells and a variable amount of extracellular polymeric substances (EPS) that function as an impermeable protecting coat that limits the diffusion of chemical substances, leading to recurring infections and resistance to antifungals. Biofilm formation in *Candida* species shares four steps: (1) adhesion (binding of yeast cells to the surface); (2) proliferation (development and multiplication of the adhered cells, which are concomitant with a morphological change and secretion of hydrolytic enzymes); (3) maturation (formation of layers of different types of cells); and (4) dispersion (detachment of cells that disseminate in the medium through the bloodstream) (Figure 1) [19]. The ability of *Candida* species to form biofilms largely depends on its ability to successfully grow both on biotic and abiotic surfaces forming cell associations that hinder their treatment with antimycotic agents and facilitate their propagation [20,21]. Treviño-Rangel et al. reported that patients with candidemia caused by biofilm-forming strains had a 70% mortality rate compared to 45.7% in patients infected with non-biofilm-forming strains [22].

*C. albicans* is a member of the healthy human microbiota, asymptomatically colonizing several niches in the body, including but not limited to the gastrointestinal (GI) tract, female reproductive tract, oral cavity and skin [21]. In most individuals with a healthy immune system, *C. albicans* is a harmless commensal that exists in harmony with other members of the microbiota. However, disturbances to this delicate balance, resulting, for example, from variations in the local environment (pH shifts or nutritional changes), the use of antibiotics or alterations in the immune system (caused by an infection or immunosuppressant therapy), can enable *C. albicans* to rapidly proliferate and cause infection. Further characterization of the targets of Bcr1 has highlighted the major contribution of cell surface proteins to biofilm formation, among which are the hyphal adhesins Als1, Als3 and Hwp1, which appear to establish heterotypic interactions that are necessary for maintaining cell–cell contacts within the biofilm [23,24]. This network comprises four “master” transcriptional regulators, Ndt80, Brg1, Rob1 and Efg1 [25]. *C. albicans* strains defective in Efg1 only form pseudohyphae on solid media and do not germinate at all in liquid media. The Efg1 regulator protein is a key factor for the formation and subsequent development of *C. albicans* biofilms [26]. Bcr1 and Tec1 [27] were found to be both required for normal biofilm development as tested both in vitro, under standard laboratory conditions, and in vivo, in rat catheter and rat denture models [25].

Since its discovery in 2009, the emerging fungal pathogen *Candidozyma auris* (*C. auris*) has become a serious global health threat. It is frequently reported in association with nosocomial outbreaks and is of urgent concern for public health authorities. *C. auris* outbreaks are characterized by the persistent colonization of patient skin and abiotic surfaces, which can remain positive for extensive periods and serve as a source of contaminative transmission [28]. Unlike other *Candida* species, *C. auris* can persist and thrive in healthcare facilities. Recent reports have shown that it can form biofilms on surfaces recovered from hospital rooms during outbreaks. However, these biofilms are weaker than those produced by *C. albicans* [29]. *C. auris* biofilms were found to be composed mainly of yeast cells adhering to the catheter material, with a little ECM [30].

Studies of *Nakaseomyces glabrata* (*C. glabrata*) have revealed a thick network of yeast cells embedded in extracellular material, lacking the hyphal component seen in *C. albicans*. This raises questions about the similarities and differences in *C. glabrata* and *C. albicans* biofilm formation, as hyphal differentiation is critical in *C. albicans* biofilms [31]. Little is known about the composition of the *C. glabrata* biofilm matrix except that it includes proteins and carbohydrates, including β-1,3-glucans [32,33]. Unlike other *Candida* species, *C. glabrata* is not polymorphic, growing only as blastoconidia. Its haploid genome is a key distinguishing characteristic, unlike the diploid genome of *C. albicans* and other *Candida* species. *C. glabrata* cells (1–4 µm) are smaller than *C. albicans* (4–6 µm), *C. tropicalis* (4–8 µm) and other *Candida* species blastoconidia [34].

*Pichia kudriavzevii* (*C. krusei*) has cylindrical cells measuring up to 25 µm long. Their form is similar to long-grain rice, contrasting with other *Candida* shapes. Like *C. albicans*, they show thermodimorphism, producing hyphae at 37 °C and blastoconidia/pseudohyphae at lower temperatures [35]. *C. krusei* is capable of forming biofilms on polyethylene, polyvinylchloride and glass. These factors contribute to the development of severe infections in tissues and organs [36]. These fungal biofilms are particularly sensitive to fluconazole when generated on polystyrene surfaces [37].

The *Meyerozyma guilliermondii* (*C. guilliermondii*) complex is a genetically diverse group of yeast species, including *C. guilliermondii*, *C. fermentati*, *C. carpophila* and *C. xestobii*. The incidence of candidemia due to the complex ranges from 1 to 3%, depending on the region [38].

*Clavispora lusitaniae* (*C. lusitaniae*) was first documented in 1979 and has attracted attention because it exhibits natural resistance to several antifungals. It has been responsible for various types of candidiasis in patients with cancer and other diseases; it has also been associated with peritonitis and meningitis [39].

*C. parapsilosis* is the major non-*C. albicans* species involved in the colonization of central venous catheters, causing bloodstream infections. *C. parapsilosis* produces colonies of different shapes. Most isolates have stable phenotypes, but occasional switching has been observed. Elongated cells (pseudohyphae) are more common amongst irregular non-smooth morphotypes, which also form biofilms better [40]. Biofilm-forming *Candida* BSIs have been associated with the highest hospital mortality [41].

*C. tropicalis* was the most prevalent species among the biofilm-forming organisms (67.5%) in candidemia, even more so than *C. albicans* (30.3%). *C. tropicalis* forms a biofilm in the model of a dense network of yeasts and pseudohyphae (hyphae in some strains) surrounded by an ECM with a low content of carbohydrates and proteins. In comparison to other medicinally important *Candida* species, *C. tropicalis* cells are relatively large (4–8 × 5–11 µm) [42].

### 2.2. Aspergillus *spp.*

**Figure 2:**
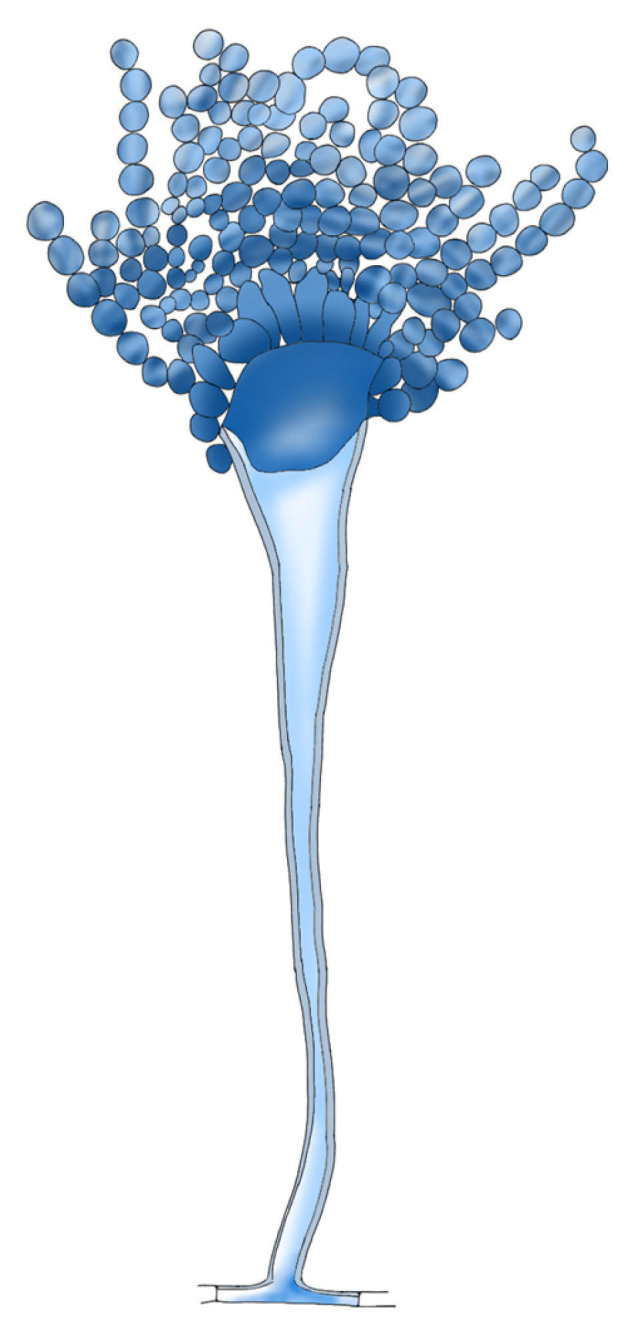
*A. flavus* structure.

*Aspergillus* spp. is a saprophytic, filamentous fungus that grows ubiquitously in soil. *Aspergillus* spp. causes a wide range of diseases in plants, birds and mammals. *A. fumigatus*, *A. terreus*, *A. flavus* and *A. niger* are the main agents that cause opportunistic infection in humans, but approximately 90% of infections are caused by *A. fumigatus*. Those species cause diseases, from primarily asymptomatic aspergillomas to invasive pulmonary aspergillosis (IPA). Aspergillomas are ball-like structures of fungal mycelia that form biofilm, extracellular matrix and host immune cells that form in preexisting lung cavitations [43]. *Aspergillus* spp. are able to produce a thick biofilm rich in extra cellular matrix (Figure 2). The ECM is composed of polysaccharides, hydrophobins, melanin and environmental DNA (eDNA) to protect its own mycelium from adverse external factors, such as heat shock, oxidative stress and nutritional deficiencies [44]. The various species of *Aspergillus* produce biofilms with different compositions.

*A. fumigatus* biofilm matures in 24 h. At the final stages of growth, the biofilm becomes an intricate network of filaments with a dense secret ECM and regions of limited oxygen. The eDNA is very important in this structure, conferring a more solid and resistant structure but also a reservoir of genes. This makes both successful immune cell responses and antifungal treatment difficult [45].

*A. flavus* biofilm happens between 24 and 48 h: after 24 h, the biofilm is multilayered; at 48 h, the presence of water channels and minimal exopolymeric substance production is visible; and after 72 h, conidial heads and abundant conidia were observed, indicating the onset of the dispersion phase [46]. The ECM of *A. flavus* biofilms is mainly composed of carbohydrates and proteins, with additional contributions from lipids and nucleic acids [46].

**Figure 3:**
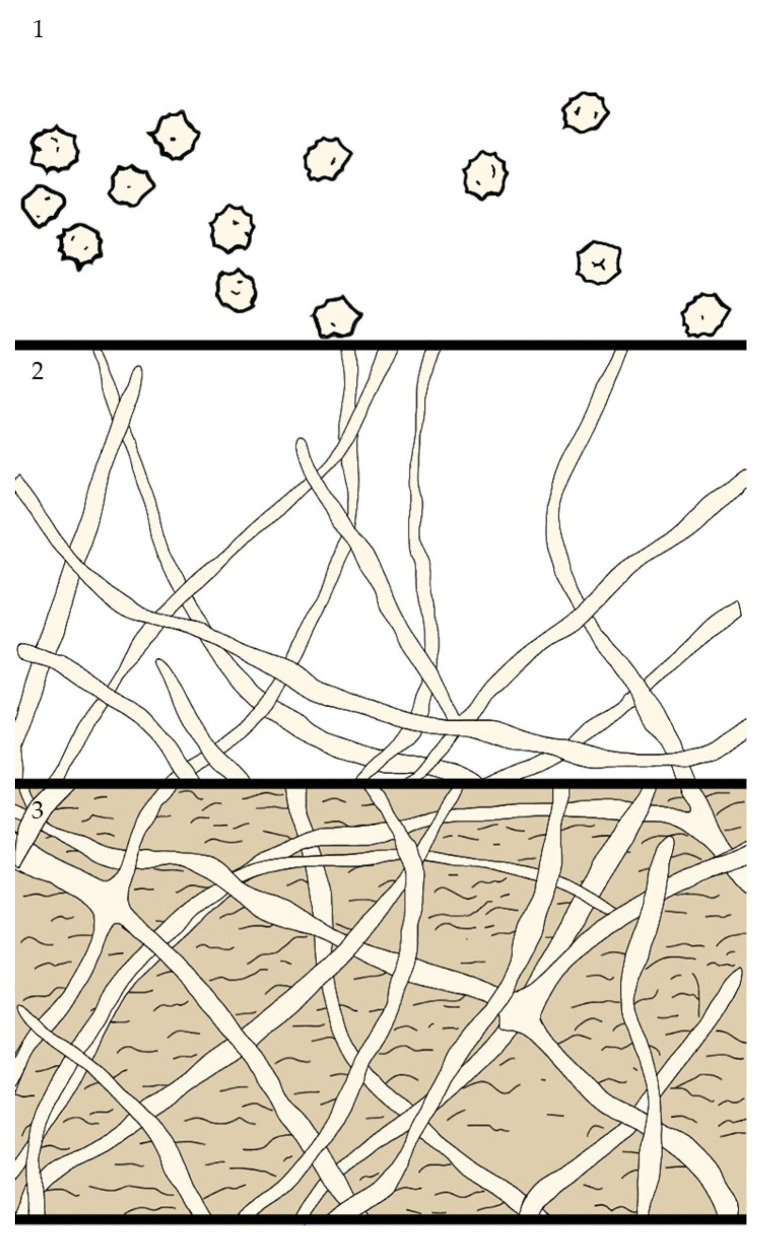
Steps of *Aspergillus* spp. biofilm formation. (**1**) Adhesion; (**2**) proliferation; (**3**) maturation.

*A. niger*’s biofilm adhesion of conidia was observed at 4 h of incubation, and after 8 h of incubation, conidial germination was initiated. After 12–20 h, the third stage of development involves apical elongation and hyphal branching, leading to monolayer formation by producing an ECM. At the fourth stage (24–36 h), the ECM appears condensed, and the conidia were extended to form hyphal network glued together (Figure 3). Mature biofilm is characterized by aerial growth of hyphae and formation of fruiting bodies. The final stage of biofilm (60–72 h) involves the disintegration of ECM and dispersion of spores [44].

### 2.3. Cryptococcus *spp.*

*Cryptococcus* spp. Is a genus of basidiomycetous fungi containing more than 30 species. The pathogenic species are two: *C. neoformans*, which cause cryptococcosis in both immunocompetent and immunocompromised hosts, and *C. gattii*, which is primarily a pathogen in immunocompromised patients [47]. Concerning *C. neoformans*, a variant where the pathogen enters the body directly through skin injury has been identified recently [48]. *C. neoformans* cryptococcosis is a very serious disease, with mortality ranging from 41% to 61%, especially in patients with HIV infection [5]. The main component of the ECM of the *C. neoformans* biofilm is the capsular polysaccharide glucuronoxylomannan (GXM). During biofilm formation, fungal cells release GXM into the surrounding environment, contributing to cell cohesion and surface adhesion [14]. Acapsular mutants, which lack the polysaccharide capsule, are unable to form biofilms, highlighting the importance of GXM in this process [49]. In addition to GXM, the biofilm matrix contains other polysaccharides and proteins, such as galactoxylomannan and mannoproteins, which contribute to the structure and function of the biofilm [14]. The formation of *C. neoformans* biofilm occurs through several stages: initial adhesion (2–4 h); after 4–16 h, cells proliferate and form evenly distributed microcolonies on the surface; at 24–48 h, the biofilm develops a complex structure with an abundant ECM. Full maturation is typically observed after 48 h [50].

### 2.4. Fusarium

*Fusarium* species (order *Hypocraeles*) are well known as plant pathogens but can cause a broad spectrum of superficial infections, such as keratitis and onychomycosis, and invasive fusarioses in humans. Twelve species are associated with infection: *Fusarium solani* (∼50% of cases) and *Fusarium oxysporum* (∼20% of cases) are the most frequent agents [51]. The fungal biofilm ECM contains immunomodulatory signaling molecules, such as hormones, growth factors, proteins and genetic material, all of which contribute to establishing biofilm-related diseases [52].

*F. solanii* biofilms forms through stages: in the first 8 h, adhesion happens; after that, conidia begin to germinate, and hyphae elongate; at 24 h, an ECM begins to accumulate. Epifluorescence microscopy with specific fluorochromes revealed that the ECM of the *F. solanii* biofilm comprises carbohydrates for structural support, proteins involved in adhesion and enzymatic functions, and environmental DNA (eDNA) that contributes to the stability and integrity of the biofilm. The presence of these components indicates a complex ECM that plays a crucial role in the biofilm’s resilience. After 48 h, the biofilm is mature [53,54].

*F. oxysporum* biofilm follows the same formation pattern as *F. solanii* and presents with the same composition. Lipids in the ECM are accumulated rapidly in the first 12 h of formation. After 48 h, newly formed cells are dispersed to start a new adhesion cycle [53].

### 2.5. Malassezia *spp.*

The genus *Malassezia* includes a group of lipophilic and most of them lipid-dependent yeasts, recognized as members of the normal skin mycota of humans and other ho-moeothermic organisms with great importance [55]. Eighteen species have been identified: ten species (*M. restricta*, *M. globosa*, *M. arunalokei*, *M. sympodialis*, *M. dermatis*, *M. slooffiae*, *M. furfur*, *M. obtusa*, *M. japonica* and *M. yamatoensis*) have mainly been isolated from human skin, whereas the others are normally isolated from animal skin [56].

*M. sympodialis* and *M. furfur*, the most common species isolated worldwide from healthy skin, have also been associated with various human skin disorders [57,58]. Although it is considered the etiological agent of pityriasis versicolor, since *Malassezia* lives on the skin surface, it is usually associated with certain dermatoses, such as seborrheic dermatitis/dandruff, atopic dermatitis and psoriasis, among others [59].

*Malassezia* species are considered important emerging pathogens due to their ability to cause superficial and systemic infections in both immunocompromised and immunocompetent hosts. Studies have shown how this fungus colonizes or infects by interacting at a molecular level with the host tissues and components of the immune system [60]. In addition, *Malassezia* species have been associated with systemic infections such as catheter-acquired sepsis, peritonitis, fungemia and pulmonary infection in patients receiving lipid parenteral nutrition [61,62].

### 2.6. Dermatophytes: Tricophiton, Microsporum and Epidermophiton

Dermatophytes are pathogens that exhibit a strong tropism for keratin-rich tissues, such as skin, nails and hair. They can infect both humans and animals, and they are also present in soil, where they utilize decomposing keratinous materials as a nutrient source. According to the latest taxonomy proposed by de Hoog et al., dermatophytes are currently classified into seven clades, each corresponding to one of the following genera: *Trichophyton*, *Epidermophyton*, *Nannizzia*, *Paraphyton*, *Lophophyton*, *Microsporum* and *Arthroderma* [63]. The first studies on dermatophyte biofilms date back to the early 2000s. In 2002, Burkhart et al. suggested that the formation of a dermatophytoma, a localized fungal mass within the nail, could indicate the ability of dermatophytes to form biofilms [64]. However, to date, only a limited number of studies have investigated dermatophyte biofilms, particularly regarding morphological and compositional differences across genera and species. It has been reported that *Trichophyton rubrum*, *T. tonsurans* and *Microsporum gypseum* produce denser biofilms with greater biomass and ECM production [65]. *T. rubrum* has also been shown to produce higher biomass when grown on nail fragments compared to standard in vitro conditions [66]. In contrast, *Trichophyton mentagrophytes* and *Microsporum canis* tend to form weaker biofilms [67]. Through scanning electron microscopy (SEM) analysis, the biofilms formed by *M. canis* were characterized by a complex, three-dimensional organization of the mycelial network, with hyphal filaments extending in various orientations. Additionally, a polysaccharide-enriched ECM was identified, enveloping segments of the hyphal structures and facilitating interconnections among hyphae. Although regions of the matrix exhibited dense organization, it was predominantly observed as a thin and porous layer, primarily distributed across the superficial zones of the mycelium [68].

## 3. Biofilm Quantification

**Figure 4:**
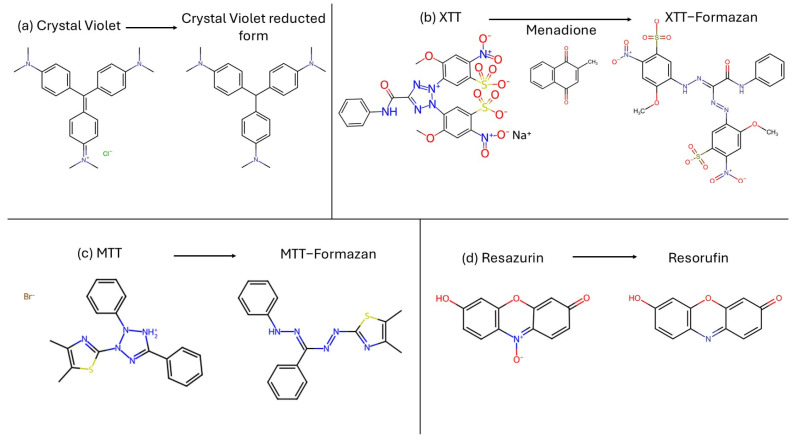
Chemical reaction carried out to measure biofilm. (**a**) Crystal violet reduction assay; (**b**) XTT-menadione reduction assay; (**c**) MTT reduction assay; (**d**) Resazurin reduction assay.

Biofilm quantification can be carried out using different types of measurement, biomass coloration and reduction assay to determine the metabolic active cells (Figure 4), or it can be measured by microscopy analysis (e.g., SEM, TEM, CLSM) or by a colony counting. Here, we describe the most used methods for fungal biofilm determination.

### 3.1. Colorimetric Methods

Crystal violet (CV) [tris [4-(dimethylamino)phenyl] methylium chloride], also known as Gentian Violet, is a triarylmethane synthetic dye. It is used to measure biofilm by staining the total fungal biomass [69]. Alternatives to measurements of total biomass like CV include assays that color only vital cells, like XTT-menadione assay, MTT assay and resazurin assay.

XTT [2,3-bis(2-methoxy-4-nitro-5-sulfo-phenyl)-2H-tetrazolium-5-carboxanilide] is a tetrazolium salt. The colorimetric changing reaction is carried out with auxilium of menadione, an electron acceptor, and it is a direct correlation of the metabolic activity of the biofilm. The intensity of color changing must be measured with a spectrophotometer [70,71,72].

MTT, 3′-(4,5-dimethylthiazol-2-yl)-2,5-diphenyl tetrazolium bromide, is a yellow tetrazolium salt used to determine cell proliferation. Living cells dehydrogenase enzymes transform MTT in formazan, an insoluble product colored purple. The intensity of the color is read by a spectrophotometer [73,74,75].

Resazurin, 7-hydroxy-10-oxidophenoxazin-10-ium-3-one, is a cell-permeable redox indicator that can be used to monitor viable cell numbers. This non-fluorescent blue dye is converted into pink-fluorescent resorufin in the presence of metabolically active cells. NADPH dehydrogenase is probably responsible for the reduction. This conversion can be detected through the visual observation of its pink color or absorbance readings of the ratio of resorufin/resazurin [73,76].

### 3.2. Colony Counting

After the incubation time, the planktonic cells are removed from the plate’s well and the adherent cells are scraped off using pipette tips and resuspended. The cell suspension is serially diluted (10–8 dilution) and spread on a plate with solid growth medium. After incubating the plates, the number of CFUs is counted on the surface [77].

### 3.3. Microscopic Quantification (SEM, TEM and CLSM)

Confocal laser scanner microscopy (CLSM) enables the creation of detailed 3D images by collecting a series of optical sections of the sample. This involves sequentially scanning different focal planes at various depths, eliminating out-of-focus light using a pinhole. The images from each plane are then digitally reconstructed to form a 3D model of the sample. Fluorescence-CLSM has been extensively used to study dispersions of fluorescein isothiocyanate (FITC)- and rhodamine isothiocyanate (RITC)-labeled silica spheres [78]. Some nanoparticles are naturally fluorescent without the need to dye them; with this, it is also possible to distinguish the particles that are at a surface–surface separation smaller than the resolution of the microscope, provided the particles contain the dye within their core, which is surrounded by a nonfluorescent shell.

Transmission electron microscopy (TEM) uses a high-voltage electron beam to image ultra-thin specimens. The electron beam passes through the sample, which must be partially transparent to electrons. Scattered and transmitted electrons are focused by lenses to form a magnified image, which can be observed on a fluorescent screen or captured using CCD cameras. TEM can also be used for electron diffraction, which helps analyze crystal structures. Z-contrast imaging (e.g., via high-angle annular dark field detectors) provides atomic resolution based on atomic number.

Limitations include sample damage due to radiation (especially in hydrated or soft matter), sample thickness limited to a few hundred nanometers and low signal-to-noise ratio in cryo-samples.

Recent advances allow for TEM operation at higher pressures and flexible temperatures. Cryo-electron tomography enables 3D imaging of macromolecular complexes, achieving resolutions of 5–20 nm.

Scanning electron microscopy (SEM) scans a focused electron beam across the surface of a solid sample, collecting emitted electrons to produce an image. It provides topographic contrast (via secondary electrons) and material contrast (via backscattered electrons). The images appear quasi-3D due to the high depth of focus. This technique requires minimal sample preparation and is good for imaging large samples. The disadvantages of this method are its long exposure time and lower resolution than TEM due to the lower electron energies.

## 4. Nanoparticles Strategies

**Figure 5:**
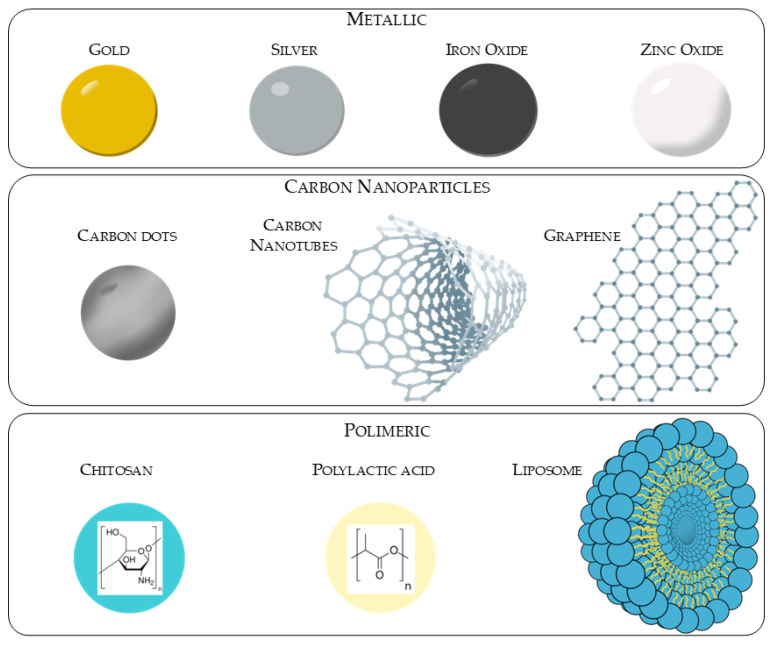
Representation of different types of nanoparticles.

According to ISO 80004-1:2023 [79], a nanomaterial is defined as a tiny particle with a size ranging between 1 and 100 nanometers (nm) (Figure 5), which can be found in nature (e.g., dust) or produced artificially. They can be produced using three different methods: physical, chemical or biological. These methods can be organized in two different groups depending on the route they follow: top-down (physical method) and bottom-up (chemical and biological method) [80]. Once synthesized through one of these two major routes, the resulting nanoparticles should be characterized in terms of their composition, size, electric surface charge and surface morphology using methods such as dynamic light scattering (DLS), scanning electron microscopy (SEM), transmission electron microscopy (TEM), infrared (IR) and X-ray diffraction (XRD) [81]. A number of these nanoparticles are currently available on the market in the capacity of medical devices or therapeutic alternatives. Illustrative examples will be provided in the corresponding paragraphs.

### 4.1. Metallic Particles (Au, Ag, Fe_3_O_4_, ZnO)

The surface-to-volume ratio of nanosized metallic particles is a key factor that alters the physical, chemical and biological properties of these particles. The exploitation of these properties has led to the use of nanoparticles in a variety of applications [82]. Metal nanoparticles can be synthetized by the two different routes as defined previously. The first physical route utilizes methods such as evaporation, condensation and laser ablation. The second method is the chemical or biological method, whereby metal ions are dispersed in a solution and reduced to form aggregates [83]. Gold and silver, also known as noble metals, show great promise in the form of nanoparticles, which is attributable to their distinctive properties and versatile applications, including their potential as antimicrobial agents [81]. Metallic nanoparticles can be formed with one, two (bimetallic), three (trimetallic) or more metals [84]. The main mechanism behind the efficacy of metallic nanoparticles against biofilms is the generation of reactive oxygen species (ROS). ROS disrupt biofilm ECM, aiding in the deeper penetration of the nanoparticles, but also damage lipids, proteins and DNA, leading to cell apoptosis or necrosis [85,86,87].

- **Gold**: Gold (Au) is a transition metal recognized as biocompatible. In recent years, the advent of nanotechnology has led to a re-evaluation of gold as a potential element for use in the synthesis of nanoparticles for medicinal applications [88]. The chemical–physical properties of AuNPs, as well as their biocompatibility with the body, have made them of considerable importance in pharmacological research for applications such as anti-rust, anti-bacterial, anti-viral, drug delivery and anti-tumor activities [89]. They can also be linked with biological molecules to better exploit their functions [90,91,92]. The in situ chemical synthesis of AuNPs involves reducing gold salts and subsequently stabilizing the NPs. The most prevalent chemical syntheses are those delineated by Turkevich–Frens and Brust–Schiffrin. Turkevich described a method for synthesizing AuNPs by reducing hydrogengentetrachloroaurate (III) (HAuCl_4_) with trisodium citrate, which also acts as a stabilizer. Frens, meanwhile, described the ratio of reagents needed to control the size of the nanoparticles [93]. The Brust–Schiffrin method involves transferring Au^3+^ from an aqueous phase to an organic phase (toluene), followed by its reduction using NaBH_4_. The formation of gold nanoparticles is evidenced by a change in the color of the organic solution [81]. Aurimmune^®^ (CYT-6091, patent number US8007790B2 [94]), developed by CytImmune, uses gold nanoparticles coated with PEG and TNF-α for tumoral targeting. This technology was tested in phase I clinical studies.
- **Silver**: Silver has long been recognized for its disinfection properties and therapeutic applications, with its use preceding the advent of antibiotics [95,96]. In the context of advancing nanotechnology, silver has once again become the focus of research, with studies examining its antibacterial and antifungal properties [97]. Silver nanoparticles prepared using biological synthesis demonstrate high stability and efficiency as antimicrobial agents [82,98,99]. The chemical synthesis of AgNPs typically uses three main components: metal precursors (AgNO_3_ solution), reducing agents and stabilizing or coating agents. The reducing and capping agents can be chosen to achieve the desired characteristics of the particles (e.g., nanoparticle size, distribution, shape and dispersion rate) and can be chemical compounds, essential oils or extracts from plants or microorganisms. Due to their high yield, chemical methods are preferred to physical methods. There has been growing interest in the biological syntheses of nanoparticles, as they are cost-effective, reliable and environmentally friendly methods that aim to mitigate the risks associated with using of certain chemical reagents [81]. Acticoat^®^ (Patent: US20040001880A1 [100]), by *Smith & Nephew*, is an antimicrobial wound dressing that utilizes nanocrystalline silver created to slowly release Ag^+^ ions to prevent bacterial infection.
- **Iron Oxide**: Iron oxide is a class of compounds formed by the oxidation reaction between iron and oxygen. The most notable compounds in this category are magnetite (Fe_3_O_4_), a mineral with strong magnetic properties that is composed of Fe (II) and Fe (III), and hematite (Fe_2_O_3_) [101]. These nanoparticles are widely used in many different biomedical applications. They can be employed in magnetic resonance imaging (MRI) and magnetic particle imaging (MPI), as well as for the targeted delivery of biological molecules (e.g., protein or antibodies). They can be also used to detect tumors or metastases in different types [102,103]. While the top-down method is elaborate and does not allow the particle size to be determined, the bottom-up approach only requires common and cheap reagents: ferric or ferrous salts and sodium borohydride [104,105]. A non-toxic reagent, such as an aqueous extract from a plant or microorganism, can be used as a substitute for sodium borohydride [104,105]. Physical procedures are characterized by their complexity and the inability to control the size of particles in the nanometer range. In contrast, the chemical method is a process that can be varied in terms of shape, size and composition. These factors dependent on the pH level, the composition and type of salt used. The coprecipitation of Fe^2+^ and Fe^3+^, with the concomitant addition of a base, is the process by which iron oxides can be synthesized [106]. NanoTherm^®^ by *MagForce AG* (approved in Europe with patent number EP1871423B1 [107]) utilizes iron nanoparticles. After injecting them into the tumor, an alternating magnetic field is used to generate localized heat and locally treat glioblastoma and prostate cancer.
- **Zinc Oxide**: Zinc is an essential element found in human tissues, where it plays a vital role in regulating various biological processes, such as including cellular homeostasis, protein synthesis, enzymatic reactions and the immune response [108,109]. Zinc oxide is used across a wide range of industrial sectors around the world. In the pharmaceutical industry, its notable properties include antimicrobial, wound-healing and anticancer activities, contributing to its use in various therapeutic areas. In cosmetics, it is used in sunscreen formulations due to its ability to scatter ultraviolet radiation. It is also used in water purification [110,111,112]. The Food and Drug Administration (FDA) recognized ZnO as a generally recognized as safe (GRAS) substance [113]. As with other metal nanoparticles, ZnO-NPs can be synthesized in two ways: by the top-down method, which involves mechanical milling, ablation or sputtering, or by the bottom-up method, which includes physical, chemical or biological synthesis. In both cases, utilizing a capping and stabilizing agent is essential [114,115]. Zinc oxide nanoparticles are used for broad-spectrum UVA/UVB protection in FDA-approved technologies like ZinClear™ by Antaria Ltd. and NOVA Minerals (Patent code: WO2020118369A1 [116]).

### 4.2. Polymeric

- **Chitosan**: Chitosan is a biocompatible cationic polymer consisting of a straight chain that is produced by the partial deacetylation of chitin. It is classified as a GRAS substance by the FDA. It is formed by the linkage of glucosamine and N-acetyl glucosamine through a 1,4-glycosidic bond. It is a principal component of fungal cell walls, but it has also been detected in the scales of insects and fish [117]. Chitosan nanoparticles (CNPs) are materials with distinctive physicochemical properties. They are biocompatible and biodegradable and have low toxicity. Simple to prepare, they have a wide range of applications in medicine, biomedical engineering, agriculture, food and the pharmaceutical industry. Applications include drug delivery, advanced cancer therapy and biological imaging and diagnosis [118]. The positive surface charge exhibited by CNPs makes them intrinsically stable within the human body, making them an optimal delivery system for medical applications [119]. Due to their amino groups, CSNPs, derived from chitin, carry a positive charge. This allows them to interact electrostatically with the negatively charged fungal cell membranes, causing increased membrane permeability, disruption of the cell wall and leakage of intracellular components, leading to fungal cell death [120,121]. NanoChit^®^ is composed of chitosan nanoparticles combined with plant extract and is used for skin whitening and anti-aging creams. It is sold as a cosmetic ingredient with INCI registration in Europe and Korea.
- **Liposome**: Liposomes are spherical lipid vesicles composed of lipid bilayers that contain an aqueous phase in which drugs can be dissolved. The bilayer of liposomes may be composed of natural or synthetic phospholipids, which determine the final properties of the liposomes [122]. The spontaneous closure of the liposome bilayer is due to the hydrophobic groups that constitute the phospholipids. During liposome formation, drugs can be loaded into the aqueous phase or within the membrane using various techniques [123]. Using liposomes as drug carriers has a number of benefits, including modulating release within the body, enhancing solubility, mitigating the toxicity of certain drugs and augmenting their activity [124]. Liposomes can be categorized according to their bilayers into distinct types: large unilamellar vesicles (LUV), small unilamellar vesicles (SUV) and multilamellar vesicles (MLV, comprising multiple concentric vesicles, or MVV, comprising multiple enclosed vesicles within a single vesicle) [125,126]. Liposome can deliver the transported drug directly inside the fungal cell. They can cross both cellular wall and membrane through various mechanisms including lipid exchange, surface interactions, fusion, endocytosis and pinocytosis [127,128]. Liposome nanoparticles loaded with Amphotericin B, AmBisome^®^ (patent US5965156A [129]), are already approved by the FDA and EMA for the treatment of systemic fungal infections and leishmaniosis [130].
- **Polylactic Acid**: Polylactic acid (PLA) is a biopolymer derived from lactic acid, which can be produced from sugar cane or corn. When this biopolymer degrades, it produces non-toxic reaction products, including water, carbon dioxide and lactic acid, the starting monomer. The compatibility of these molecules with the human body means this polymer can be used to produce suture threads and for the controlled release of drugs or vaccines [131,132,133]. Polylactic acid nanoparticles can be synthesized using a variety of methodologies, including emulsion, precipitation and in situ through spray-drying techniques [134,135]. PLA nanoparticles enter cells primarily via clathrin-mediated endocytosis; once inside, these nanoparticles are able to avoid degradation pathways, thereby releasing their cargo directly into the cytoplasm [134,136]. Lupron Depot^®^ (patent US8921326B2 [137]) by *AbbVie* uses polylactic acid nanoparticles to deliver leuprolide acetate in a sustained way over 1–6 months and treat prostate cancer, endometriosis and fibroids.

### 4.3. Carbon Nanomaterials

Carbon nanoparticles (CNPs) are composed of carbon, an element that gives them high stability, heat conductivity, durability and biocompatibility, while keeping toxicity to the human body low. Examples of carbon-based nanomaterials include carbon dots (CDs), carbon nanotubes (CNTs) and graphene. Depending on the type of formation, CDs can be further subdivided into carbon quantum dots (CQDs), graphene quantum dots (GQDs) and carbonized polymer dots (CPDs) [138]. The photoluminescence exhibited by CDs is attributed to their size, high conductivity and low production cost. The non-toxicity and biocompatibility of CDs allow them to be used in clinical and pharmaceutical applications such as drug delivery, biosensors and bio-imaging systems [139,140]. CDs can be synthesized using a variety of methodologies, including a top-down approach involving the cleavage or chemical excision of a carbon resource, such as graphene, using lasers or ultrasound. Another type of synthesis is the bottom-up, approach, which involves separating covalent bonds in small organic molecules (e.g., glucose, citric acid and sucrose) using microwaves (microwave pyrolysis) [138,141,142]. Carbon nanotubes are cylindrical-shaped and composed of graphene layers. The classification of carbon nanotubes is based on the number of walls in single-walled carbon nanotubes (SWCNT) and multi-walled carbon nanotubes (MWCNT) in each layer. Single-walled carbon nanotubes (SWCNTs) typically possess a diameter ranging from 0.4 to 2 nanometers (nm), whereas multi-walled carbon nanotubes (MWCNTs), which are constituted by concentric layers of graphene sheets, exhibit a diameter that can extend up to 100 nm [143,144,145]. Carbon nanotubes (CNTs) have found widespread application in a variety of fields, including biomedicine, drug delivery (for example, doxorubicin) [146], diagnostics, biosensors, the conjugation and targeting of molecules and engineering [147,148]. Graphene is constituted by a single layer of carbon atoms that are linked by covalent bonds. It is the elementary constituent of other carbon materials of varying dimensions [149]. The mechanism by which carbon-based nanoparticles function is not yet fully understood. It has been established that one of the mechanisms by which these cells may act against fungal cells is through internalization via electrostatic interactions. The process of internalization can also occur through the conjugation of the nanoparticles with other molecules that can interact with ergosterol and facilitate membrane crossing. Upon passing through the cell wall, the nanoparticles proceed to generate ROS, which in turn result in cell wall damage and protein or DNA denaturation [150]. As of today, there are no products based on carbon nanoparticles formally approved by the FDA for therapeutic use in humans. Some related products have been authorized for indirect or non-therapeutic uses or are in advanced experimental phases with partial authorizations such as Investigational Device Exemption (IDE) or Investigational New Drug (IND) status.

## 5. Research Methodology

A literature review was conducted by searching articles published from 2015 up to June 2025 on PubMed and Google Scholar using term ‘nanoparticles’ in combination with ‘fungal biofilm’, ‘*Candida* biofilm’, ‘*Aspergillus* biofilm’, ‘*Cryptococcus* biofilm’, ‘*Fusarium* biofilm’ and ‘dermatophytes biofilm’. The selection of articles was based on the presence of nanoparticle characterization in the study. In the course of the present study, articles in which fungi, or their metabolites, were employed exclusively for the synthesis of nanoparticles, whilst antibiofilm action was performed on bacterial microorganisms, were excluded from the search results.

## 6. Nanoparticles on Fungal Biofilm

Research in the field of nanoparticles and their application in medicine is subject to constant updating. However, there is a paucity of research conducted on the action of these compounds on biofilms produced by fungi of medical interest. Here, we describe some recent studies that used different nanoparticle types to treat fungal biofilm (listed below in Table 1). In vivo studies of nanoparticles tested on fungal biofilm are reported below in Table 2.

### 6.1. Gold Nanoparticles

Gold nanoparticles synthetized with β-caryophyllene and free β-caryophyllene show a minimal inhibitory concentration (MIC) of 512 μg/mL and >2048 μg/mL, respectively. The concentration of β-c-AuNPs that resulted in the maximum level of initial stage inhibition of fungal biofilm in *C. albicans* was found to be 256 μg/mL [151]. Fucoidan–gold nanoparticles (Fu–AuNPs) and phloroglucinol gold nanoparticles (PG-AuNPs) were used on mixed (fungal-bacterial) biofilm. Fu–AuNPs MIC concentration results as 1024 μg/mL, while the MFC results as 2048 μg/mL. A concentration of 512 μg/mL was found to be the maximum inhibition of biofilm formation for *C. albicans* + *S. mutans* and *C. albicans* + *S. aureus*. Single and mixed mature biofilms have the maximum eradication at 2048 μg/mL [153]. Significative inhibition of the early-stage biofilm in the presence of PG-AuNPs was shown to be 256 μg/mL for *C. albicans* and 512 μg/mL for mixed early-stage biofilm of *C. albicans* + *S. aureus* [152].

Resveratrol gold nanoparticles (AuNpRSV) and gold nanoparticles (AuNp) were tested on a reference strain of *C. albicans* and on *C. albicans* isolated from a HIV patient, and they showed MIC values of 2.46 μg/mL in both of the strains. The MIC value for Resveratrol (RSV) alone for both *Candida* strains was determined to be >256.00 μg/mL. MFC values were 4.92 μg/mL for AuNpRSV and AuNp in all strains tested. AuNps significantly reduced the biofilm viability of *C. albicans* reference strain at MIC and 5× MIC. For isolated *C. albicans* strain, biofilm viability was reduced only at 5× MIC in the presence of AuNp. AuNpRSV significantly decreased the viability of *C. albicans* reference and isolate at MIC concentration. These results demonstrate the major effects of Resveratrol-coniugated gold nanoparticles in order to reduce the biofilm MIC concentration also on isolated strains [154].

The MIC values of synthesized *Crinum latifolium* gold nanoparticles (AuNPs) ranged between 250 and 500 μg/mL, while MFC values ranged between 500 and 1000 μg/mL. CLSM analysis using live/dead staining, i.e., Con-A-FITC and propidium iodide, found that biofilm formation on glass cover slips was reduced by >95% when exposed to of 50 μg/mL of AuNPs, while up to a 60% reduction was seen at 25 μg/mL of AuNPs [155].

Testing the same concentrations of AuNPs, indolicidin and AuNP–indolicidin demonstrates that gold nanoparticles funcionalizated with indolicidin forms a highly potent complex for preventing of biofilm formation and eradicating of both *Candida* ATCC and clinical isolates compared to AuNPs and indolicidin alone. The lower activity of AuNPs compared to the indolicidin suggest that the gold nanoparticles enhance the effect of indolicidin rather than eradicating of biofilm itself [156].

Chitosan–Tyrosol–gold nanoparticles (Chi-TY-AuNPs) have a MIC_80_ value of 200 μg/mL and 400 μg/mL on *C. albicans* and *C. glabrata*, respectively. The MFC value for both *Candida* species was 800 μg/mL. The BIC_80_ values of Chi-TY-AuNPs against both *Candida* spp. were 200 and 400 μg/mL, respectively. The biofilm eradicating efficacy of Chi-TY-AuNPs was found to be equal against both *C. albicans* and *C. glabrata* biofilms. The BEC_80_ value was found to be 800 μg/mL for both species [157].

### 6.2. Silver Nanoparticles

Green synthesized nanoparticles are the most studied. Silver nano particles produced with *Erodium glaucophyllum* extract have shown a MIC of 50 μg/mL against *C. albicans*, which is half the concentration of AMB. Biofilm assays revealed a significant reduction in fungal burden by 52% after treatment with EG-AgNPs at MIC concentration [158]. The antimicrobial activity of green synthesized silver nanoparticles in the presence of *Encephalartos laurentianus* leaf extract (ELLE) were tested. The MIC values of AgNPs against 13 *C. albicans* isolates ranged from 8 to 256 μg/mL. The AgNPs at a concentration of 0.5 MIC reduced the percentage of biofilm formation in all the isolates tested, and by 69.23% to 30.77% for the *C. albicans* isolates with the maximum production [159]. Antifungal susceptibility testing of silver nanoparticles on eight clinical isolates of *C. auris* under planktonic conditions showed significant antimicrobial activity. The MIC of AgNPs was <6.25 μg/mL. Dose–response curves indicate that AgNPs displayed remarkable antibiofilm effects against *C. auris* isolates. The calculated IC_50_ values ranged from 0.7 to 3.2 μg/mL, and five out of eight strains were less than 2 μg/mL [160]. Scanning electron microscopy (SEM) images of cells of *C. albicans* cells after the treatment with AgNPs derived from *A. variabilis* showed deep wrinkles and deformation. AgNPs at a concentration of 1/2MIC and MIC (6.25 and 12.5 μg/mL) also suppressed *C. albicans* biofilm development and decreased in the density of biofilm in a concentration dependent manners [161]. Another study examined the activity of two different biosynthesized AgNPs: dispersions–small monodisperse AgNPs (mAgNPs) and larger polydisperse AgNPs (pAgNPs), both of which were prepared using *Vitis vinifera* cane extract. Furthermore, the possibility of exploiting the synergistic effects of the plant extract (resulting from the biosynthesis process) and the NPs was investigated. *V. vinifera* cane extract alone did not inhibit the growth of the planktonic cells of *C. albicans* planktonic cells; instead, both biosynthesized silver NPs displayed significant inhibitory activity against *C. albicans*. Monodisperse nanoparticles significantly inhibited the growth of the microorganisms at concentration of 20 μg/mL and above. The presence of *V. vinifera* extract reduced the effect of the AgNPs. The highest concentration of the extract (2% (*v*/*v*)) inhibited the metabolic activity by 32% relative to the control. Interestingly, the nanoparticles had an increased inhibitory effect when used in combination with the *V. vinifera* extract as a medium. Polydisperse AgNPs were more effective at inhibiting the activity of biofilm cells, similarly to their effect on planktonic cells. The highest decrease in metabolic activity (80% relative) was observed at a concentration of 20 μg/mL pAgNPs/e [162]. Biogenic silver nanoparticles (PchNPs) from the fungus *Phanerochaete chrysosporium* were also tested and revealed antibiofilm activity, reducing *C. albicans* biofilm biomass by 80% [163]. Biogenic silver nanoparticles synthetized from *Eucalyptus camaldulensis* extract significantly inhibited biofilm formations during the initial 2 h phase and after 8 h. *C. albicans* did not exhibit a hyphal form upon treatment with 2 μg/mL bio-AgNPs; only budding or single yeast forms within a loosened structure were observed [164]. Bimetallic (Ag-Ni) nanoparticles synthesized using an aqueous extract of *Salvia officinalis* leaves acted as an anti-biofilm agent at a concentration of 3.12 μg/mL against fluconazole-resistant *C. albicans*. Introducing 1.56 g/mL of Ag-Ni NPs to the biofilm significantly altered its structure, consisting primarily of yeast cells and being devoid of either true hyphal or pseudohyphal structures [165].

The dimensions of a nanoparticle are crucial to its activity. Three different sizes of citrate-stabilized nanoparticles were tested: the diameter of AgNP-I was between 5 and 12 nm in diameter, AgNP-II was between 15 and 50 nm, and the size of AgNP-III nanoparticles was around 35–90 nm. The potential antifungal effect of the nanoparticles was tested against *C. albicans*, *C. dubliniensis*, *C. krusei*, *C. parapsilosis* and *C. tropicalis*. All three AgNP specimens inhibited the growth of the examined strains but to a different extent: AgNP-I was the most effective one, while AgNP-III exhibited the least toxic activity. The effect of these nanoparticles on *Candida* biofilm was evaluated at a concentration of 75 and 37.5 μg/mL. In most cases, the repression of biofilm development was observed to be both dose- and size-dependent. The smallest average diameter nanoparticles (AgNP-I) hampered the biofilm formation more efficiently than the AgNP-II or AgNP-III solutions. However, significant biofilm degradation could not be achieved using any of these AgNPs [166]. Silver NPs, as well as their precursor, were tested against nine species of *Candida* to evaluate the effect of the difference size on their activity. Both the ATCC and clinical isolates pathogens showed sensitivity to different concentrations of AgNO_3_ precursor in direct contact. The cationic polymer, PEI, was highly toxic to the fungi, even at the lowest concentration. The smallest nanoparticles had a lower MIC (0.078–0.625 μg/mL). Silicone catheter (SR) fragments coated with AgNPs (dimensions from 3 to 7 nm) were incubated with different *Candida* isolates to observe the biofilm structure and morphology. The results showed a reduction in the amount and extent of the biofilms, as well as changes in cell morphology and structural compaction. However, the inhibition of the biofilm was not observed for all the species, which is probably related to a difference in the biofilm molecular compositions of the strains [167]. Biogenic silver nanoparticles (AgNPs) fabricated using the supernatant of *Penicillium fimorum* showed a MIC of 4 μg/mL. Their results show that at concentrations of 4 and 16 μg/mL, there is a clear inhibition of the biofilm formation of at least 80% and near the result of amphotericin B [168]. In another study, *Terminalia catappa* leaf extract (TCE) was used to optimize TCE-capped silver nanoparticles. After 24 h of treatment with concentrations of 0.95, 1.95, 3.90 and 7.80 μg/mL, the inhibition of *C. albicans* biofilm formation was, respectively, 48.70%, 50.23%, 57.76% and 64.41% [169]. Pathogenic fungi such as *Aspergillus fumigatus* can also be used as a promising biomass for the green synthesis of biogenic silver nanoparticles. The percentage of biofilm inhibition by the biosynthesized Ag NPs’ sub-MIC concentrations of 10 mg/mL of *A. flavus* and *C. albicans* were 75.45 and 70.25%, respectively [170].

Nanoparticles combined with another substance can create a synergistic effect. When used against resistant *C. albicans*, AgNPs combined with fluconazole appear to be more active than fluconazole alone. High MIC values (128 μg/mL) for fluconazole alone and MIC of 32 μg/mL for AgNPs alone were observed for both strains tested. The MICs for AgNPs and fluconazole combined were, respectively, 0.5 μg/mL and 0.5 μg/mL (for CAR15) and 1 μg/mL (for CA-R21) [171]. Other results confirm that the combinate use of mycogenic Ag-NP and fluconazole exhibits strong in vitro antifungal synergy against resistant clinical isolates of six *Candida* species. A lower antifungal effect was displayed when FLC (64–128 μg/mL) and Ag-NPmyc (50–100 μg/mL) were used independently. For example, in the case of *C. albicans*, the MIC of fluconazole for *C. albicans* was reduced from 128 μg/mL, when used alone, to 8 μg/mL, when used in combination with nanoparticles [172].

Microwave-irradiated kappa-carrageenan (CRG)-capped AgNps have been shown to have BIC_80_ (80% of biofilm inhibiting concentration) and BEC_80_ (80% biofilm eradicating concentration) values of ~300 μg/mL for both *C. albicans* and *C. glabrata*. The data presented indicate concentration-dependent biofilm inhibition and the eradication of pre-formed biofilms [173]. The results also indicated a potent inhibitory effect of AgNPs on *C. auris* biofilm in a dose-dependent manner. The IC_50_ was observed at 60 ng/mL. AgNPs also demonstrated efficacy when tested against preformed biofilms of the same *C. auris* strain, resulting in a calculated IC_50_ of 480 ng/mL. Ultrastructural analysis using SEM techniques on *C. auris* biofilm treated with AgNPs showed scarce cells with significant changes to the yeasts’ shape and surface appearance, from smooth to rough, indicating cell wall damage and disruption [174]. In another study, AgNPs at different concentrations of 108 μg/mL and 54 μg/mL were tested against *Aspergillus* and *Fusarium* biofilms. The AgNPs showed strong antifungal capacity, achieving up to a 99% reduction in *Fusarium* biofilm and 57% in *Aspergillus* biofilms. SEM images prove the reduction in the amount of *Fusarium* biofilm (AgNPs 108 μg/mL) [175].

### 6.3. Iron Oxide Nanoparticles

Iron oxide nanoparticles can also be synthetized using a green method. *T. indica* fruit extract was used to phyto-synthetize *T. indica*-Fe_2_O_3_ NPs, which were then tested on *C. albicans*. The observed MIC was 20.7 μg/mL and MFC was 30.8 μg/mL. Antibiofilm activity was observed to be 47% at 25 μg/mL, rising to 89% at 100 μg/mL [176]. Green synthetized CoFe_2_O_4_ nanoparticles (NPs) using *Aloe vera* leaf extract were tested by Ansari and his team. The results indicated an MIC of 1 mg/mL for *C. albicans*. CoFe_2_O_4_ NPs at concentrations of 0.125, 0.25 and 0.5 mg/mL inhibited biofilm formation by 59.8, 61.8 and 58.1%, respectively [177]. Iron nanoparticles can be used as carriers. Iron oxide nanoparticles coated with chitosan (IONPs-CS) were loaded with either miconazole or fluconazole at a concentration of 39, 78 or 156 μg/mL. These nanoparticles were then tested on a mixed preformed biofilm of *C. albicans*, *C. glabrata* and *C. tropicalis*, using MCZ and FLZ as controls. The greatest reductions in CFU numbers were achieved by MCZ, IONPs-CS-MCZ78 and IONPs-CS-MCZ156, while IONPs-CS-MCZ39 led to significant decreases in *C. albicans* and *C. tropicalis* but was unable to overcome the reductions achieved by MCZ alone. IONPs-CS-FLZ156 was the most effective compound in reducing the number of CFUs of *C. albicans* and *C. tropicalis*, the same was true for IONPs-CS-FLZ78, which surpassed the effects achieved by FLZ alone for the latter. However, no compound was able to affect the number of CFUs for *C. glabrata* [178].

### 6.4. Zinc Oxide Nanoparticles

Zinc oxide decorated with silver nanoparticles (ZnO-AgNPs) was tested against preformed biofilm by *Trichophyton mentagrophytes*. When applied to the biofilm, the ZnO-AgNPs water suspension formed spheres that remained on the surface after 24 h deposition. Suspension in a hydrophobic fluid such as polydimethylsiloxane (PDMS) was found to be more effective: 1 mL of PDMS containing 0.1031 g of ZnO-AgNPs powders was deposited in the middle of top of biofilm. After one hour, the suspension had penetrated the biofilm and after 7 days, zone of fungal inhibition could be observed [179]. ZnNPs biosynthesized by *Aspergillus fumigatus* and ZnNCs coated with the probiotic *L. salivarius* were tested against *C. albicans* isolates. ZnNCs exhibited the highest biofilm inhibition rate at 84.4%, followed by ZnNPs. *C. albicans* treated with AMB displayed a significantly lower biofilm inhibition rate than those treated with ZnNPs, probiotic *L. salivarius*, and ZnNCs [180]. Another study used lignin (L), lignin fragments (FL) and oxidized lignin fragmentes (OFL) as templates for synthesizing zinc oxide nanoparticles (ZnO NPs). The MIC_50_ concentrations of L-ZnO, FL-ZnO and OFL-ZnO NPs against *C. albicans* were 465.8, 7.31 and 31.25 μg/mL, respectively. In the presence of FL-ZnO NPs at 31.25 μg/mL, more than 80% of the biofilm formation was inhibited; at the same concentration, L-ZnO and OFL-ZnO NPs inhibited biofilm formation to a lesser extent. Biofilm inhibition was lowest in the presence of L-ZnO NPs [181]. Zinc oxide nanoparticles (ZnO-NP-B) biologically synthesized using *Lactobacillus gasseri* and two chemically synthesized ZnO nanoparticles (ZnO-NP-C1 and ZnO-NP-C2) were compared. The nanoparticles were tested against a *C. auris* strain, resulting in MIC_50_ values of 1 mg/mL, 61.9 μg/mL and 151 μg/mL, respectively. ZnO-NP-C1 and ZnO-NP-C2 demonstrated 67.9 and 51.6% inhibition of *C. auris* adhesion, respectively, effectively preventing the fungal cells from attaching to the surface. In contrast, the adhesion of *C. auris* was not measurable affected by biologically synthesized ZnO-NP-B1 [182].

### 6.5. Chitosan Nanoparticles

Phloroglucinol (PG) was encapsulated into chitosan nanoparticles (CSNPs) and tested against *C. albicans* and their mixed biofilm. MIC values against *C. albicans* were found to exceed 2048 μg/mL. Furthermore, the BMIC_80_ value of PG-CSNPs against biofilm cells was found to be higher than 4096 μg/mL. The maximum inhibition (86%) of the *C. albicans* biofilm by PG-CSNPs was observed at a concentration of 256 μg/mL. The study investigates the inhibitory effects of PG-CSNPs at a concentration of 1024 μg/mL on dual-species biofilms of *C. albicans*/*S. mutans*, *C. albicans*/*K. pneumoniae* and *C. albicans*/*S. aureus*. The results demonstrate that the maximum levels of inhibition achieved were 88.7%, 86.0% and 92%, respectively [77]. In a further study, curcumin (Cur) was loaded into chitosan nanoparticles (CSNP) and evaluated in relation to *C. albicans* and a mixed polymicrobial biofilm comprising *S. aureus*. The MIC of Cur loaded on the CSNP was found to be higher (400 μg/mL) than that of free Cur (200 μg/mL). However, both concentrations of Cur (200 μg/mL) were found to inhibit almost all mono- and polymicrobial biofilm formation. CSNP-Cur demonstrated a marginally diminished inhibitory effect in comparison to the free form of Cur [183]. The extraction of *Olea europaea* leaves was conducted in conjunction with a solution of chitosan, thus yielding the desired result of chitosan nanoparticles. It is interesting to note that CSNPs have been shown to induce a significant biofilm reduction of *C. albicans*, with a range 31.35% (±0.83) to 67.86%  (±1.19), corresponding to a concentration range of 50–1500 μg/mL of CSNPs [184]. The formation of chitosan nanoparticles (CSNP) involves the utilization of tripolyphosphate (TPP) as a cross-linking agent, followed by the loading of berberine (BBR). This process results in the production of berberine-loaded chitosan nanoparticles (BBR-CSNP). The inhibitory effects of BBR and BBR-CSNP on *C. albicans* biofilm are concentration-dependent. In the context of biofilm formation, BBR-free is demonstrably more efficacious, presumably due to the greater quantity of berberine released on mature biofilm. The effects of BBR and BBR-CSNP are reciprocal, resulting in a substantial reduction in biofilm with the utilization of chitosan nanoparticles [185]. In another study, the co-immobilization of cellobiose dehydrogenase (CDH) and deoxyribonuclease I (DNase) on positively charged chitosan nanoparticles (CSNPs) resulted in a bi-functional nanoparticle (CSNP-DNase-CDH). CDH is an extracellular hemoflavonoenzyme secreted by fungi to assist with the degradation of biomass by lignocellulolytic enzymes, while deoxyribonuclease I is an endonuclease of the DNase family. It was demonstrated that at a concentration of 100 μg/mL, both CSNP-CDH and CSNP-DNase-CDH exhibited effective biofilm inhibition; conversely, CSNP-DNase exhibited minimal effect. The results of the experiment demonstrated that the biofilms of *C. albicans* and *S. aureus* were inhibited by CSNP-DNase-CDH in a concentration-dependent manner. The MIC for 2 mM cellobiose was 88.8% and 90.5% for *C. albicans* and *S. aureus*, respectively [186]. Chitosan nanoparticles have also been found to be capable of loading natural products, such as bee venom, thereby forming BV-CSNP. Research conducted on *C. albicans* and *C. neoformans* has demonstrated MIC values of the resultant nanoparticles of 1.562 mg/mL and 3.125 mg/mL, respectively. The results obtained demonstrate levels that are higher than those observed in bee-venom-free samples, which exhibited an MIC of 6.25 mg/mL. In the experimental setting, an MIC concentration of BV-CSNP resulted in a biofilm reduction rate of 57% (*C. albicans*) and 51% (*C. neoformans*) [187]. Chitosan derivatives were prepared by incorporating salicylhydrazide into two different types of chitosan, Schiff base chitosan (SCsSB) and chitosan (SCs). The study involved the preparation of two nanocomposites, which were synthesized by combining the derivates of chitosan with titanium nanoparticles (SCs/TiO_2_-3%). The minimum biofilm inhibitory concentration against *C. albicans* was found to be 1000 μg/mL for chitosan, 500 μg/mL for SCsSB, 125 μg/mL for SCs and 7.81 μg/mL for SCs/TiO_2_-3% [188]. Another natural product loaded in chitosan nanoparticles can be carvacrol (Cv-CSNP) with different concentration ratios. The MIC of these nanoparticle types was found to be contingent upon the quantity of carvacrol employed during the synthesis process. This finding indicates that a ratio of 1:1.50 (Cv-CS) has the most effective MIC of 24 μg/mL against *C. albicans*. In contrast, other *Candida* species demonstrate a significantly higher MIC, *C. glabrata* requiring 780 μg/mL and *C. krusei* and *C. lusitaniae* requiring 1560 μg/mL, to obtain the same effect. The present study demonstrated that a reduction in biofilm formation and preformed biofilm was observed in the presence of carvacrol chitosan nanoparticles (CNP) in comparison with untreated biofilm, as well as with both chitosan (CS) and CSNP. This reduction was particularly evident in the case of *C. krusei* and *C. tropicalis*. The two species displayed a heightened sensitivity to each treatment, a response that may be attributable to variations in biofilm formation and structural characteristics [189].

### 6.6. Liposome

Anidulafungin liposomes were prepared with varying concentrations of the anidulafungin drug (1× = 0.31%, 5× = 1.55% and 10× = 3.10% *w*/*w*). These liposomes were then used on two reference strains of *C. albicans*. The three formulations showed an MIC at 12.50, 6.25 and 1.56 μg/mL for formulation 1×, 5× and 10×, respectively. These concentrations of liposomes correspond to a concentration of anidulafungin of 0.020–0.039 μg/mL (1×), 0.049–0.098 μg/mL (5×) and 0.025–0.049 μg/mL (10×). Anidulafungin liposomes were the subject of further study, which employed XTT reduction assay, to evaluate their efficacy on mature biofilm. Anidulafungin-free and untreated samples were used as controls. The results demonstrated a substantial decrease in metabolic activity in samples treated with liposome compared to the anidulafungin-free sample, which showed outcomes analogous to the untreated sample. The findings of the cell count experiment demonstrated concordance with those of the preceding experiment. The investigation revealed that the administration of anidulafungin in a free form resulted in a reduction in CFU in comparison with the control group. Conversely, the liposome formulations demonstrated a higher degree of efficacy [124].

Liposome with soy lectin (SL) and five different concentrations (0.2, 0.5, 1.2 and 5 mg) of lauric acid (LA) or myristoleic acid (MA) were formulated and subsequently tested on *C. albicans* biofilm and planktonic cell growth. It was established that formulation 2 (1 mg SL and 0.5 mg LA or MA) was the most effective, followed by formulations 3, 1, 4 and 5. An antibiofilm effect was observed; however, no such effects were observed in relation to planktonic cell growth [190].

### 6.7. Polylactic Acid Nanoparticles

Poly (L-lactide) nanoparticles loaded with ketoconazole (Keto-NP) were tested on four *C. albicans* strains. The results demonstrated a MIC range of 0.007–0.015 μg/mL for the nanoparticles, while the same strains exhibited a MIC range of 0.015–0.06 μg/mL for ketoconazole alone. Furthermore, MIC values of Keto-NP and ketoconazole were tested on four *Candida* non-*albicans* strains, with results showing 0.007 and 0.03 μg/mL for *C. dubliniensis*, 0.48 and 0.48 μg/mL for *C. krusei*, 0.03 and 0.06 μg/mL for *C. parpsilosis* and 3.90 and 7.81 μg/mL for *C. tropicalis*, respectively. The MIC was also determined for three dermatophytes: *Trichophyton rubrum* (0.015 and 0.06 μg/mL), *Trichophyton mentagrophytes* (0.06 and 0.48 μg/mL) and *M. gypseum* (1.95 and 7.81 μg/mL). These were determined for Keto-NP and ketoconazole, respectively. The antibiofilm effects of nanoparticles and free ketoconazole was measured by MTT reduction assay for all *Candida* species on mature biofilm. The study revealed that the MIC of Keto-NPs for *C. albicans* ranged from 1 to 30 μg/mL, while that of ketoconazole ranged from 3 to 27 μg/mL. These findings indicated significant intra-species variability. The results obtained exhibit a BIC_50_ of 0.05 μg/mL for ketoconazole for all three species, while a BIC_50_ concentration of 0.05, 2.5 and 0.8 μg/mL for Keto-NP was obtained for *C. dubliniensis*, *C. krusei* and *C. parapsilosis* [191].

Poly(lactic-co-glycolic) acid nanoparticles (PLNP) loaded with pterostilbene (PTB) and pomace extract were used on four *C. albicans* reference strains with the objective of determining the MIC and antibiofilm concentration. The experiments were also conducted in the presence of PTB and pomace extract alone as a control. MIC90 values for PTB and PTB-PLNP were found to be greater than 16 μg/mL for all *Candida* strains tested. The MIC of pomace extract ranged from 7.49 to 25.0 μg/mL, whilst the MIC of pomace-PLNP was 50 μg/mL. PTB-PLNP demonstrated a substantially higher level of activity in relation to biofilm formation and mature biofilm at 16 μg/mL for all strains tested in comparison to PTB alone. In both cases, compounds with pomace extract demonstrated reduced antibiofilm activity. However, significantly higher efficacy was observed for pomace–PLNP compared to pomace alone [192]. The same compounds were also tested on *A. brasiliensis*, formerly *A. niger*, with the aim of evaluating their antifungal activity in the context of biofilm formation and preformed biofilm. The results demonstrated a significant increase in activity of PTB-NP (20 μg/mL) in comparison to PTB or nanoparticles alone, with regard to both biofilm formation and preformed [193].

### 6.8. Carbon Nanoparticles

Three different types of carbon dots were synthetized: amine-rich carbon dots (CDs-NH_2_), amino and carboxyl functionalized carbon dots (Cds-CO_2_H/NH_2_) and carboxylic carbon dots (CDs-CO_2_H). MIC_50_ results demonstrate the best activity for CDs-NH_2_ on *C. albicans* planktonic cells (397 μg/mL), while CDs-CO_2_H and CDs-CO_2_H/NH_2_ have an MIC_50_ greater than 500 μg/mL. Positive-surface-charged CDs (CDs-NH_2_) showed a significant activity on biofilm formation and mature biofilm, with 89% and 95% of inhibition, respectively, at 500 μg/mL, compared to CDs-CO_2_H/NH_2_ and CDs-NH_2_. Significant results were observed for CD-NH_2_ from 125 μg/mL [141].

In order to combine the properties of carbon and metal nanoparticles, a carbon nitride matrix embedded with Ag nanoparticles was synthesized (Ag@g-CN). These nanoparticles were tested against clinical and reference *C. albicans* strains. Fluconazole and g-CN were used as controls. The results show that for g-CN MIC, values are always greater than 1024 μg/mL, while Ag@g-CN has a MIC range of 4–256 μg/mL. Ag@g-CN and fluconazole were also tested for antibiofilm activity, with fluconazole as a control. The significant biofilm inhibition was achieved with 10 × MIC Ag@g-CN, slightly higher than the results obtained with fluconazole [194]. Mixed biofilms of *C. albicans* and *S. aureus* (biofilm A) and *C. albicans* and MRSA (biofilm B) were grown on titanium discs (control) and graphene-nanocoated titanium discs (GN). The biofilms were characterized in metabolic activity (XTT), total biomass (CV) and CFU counting. For biofilm A was observed a reduction of 40% in metabolic activity, 69% in total biomass and 89% in CFU in GN disks. The reductions were, respectively, 50%, 70% and 93% for biofilm B [195]. In another study, plastic coverslips were nanocoated with graphene oxide (GO) or with graphene oxide functionalized with curcumin (GO/CU) to assess their antibiofilm potentials. The test was conducted against *C. parapsilosis* isolates. The results show inhibition of adherence with reduction in CFU by 90% for GO coating and 99.8% for GO/CU coating after 30 min. The CV assay revealed an inhibition of biofilm formation by 8% for GO coating and 64% for GO/CU coating [196].

## 7. Conclusions and Future Prospective

Estimates of fungal infections suggest a global prevalence of around 6.5 million cases, though this figure may be underestimated due to the absence of full clinical records. Research is increasingly focusing on biofilm-forming fungi and the role of biofilms in persistent infections. Biofilms hinder the effectiveness of current drugs, so alternatives with drugs, molecules, plant/microbe extracts and enzymes are being explored. Resistance to drugs due to the biofilm barrier can lead to higher antifungal drug concentrations and toxic side effects. However, nanoparticles could help overcome the biofilm barrier, allowing the eradication of pathogens at lower concentrations. Nanoparticles are used in a wide range of industries. Their composition and nature vary, so efficient preparation protocols are being researched. Most of the work on nanoparticle mass production uses green-synthesized nanoparticles. These perform well against human-pathogenic fungi, surpassing the barrier of biofilm and working synergistically with antifungal drugs. It is important to consider the limited in vivo tests carried out in the treatment of infections attributable to these pathogens, as well as stability tests, which will be indispensable before these nanoparticles can be considered as alternative treatments to those available today. It is crucial to undertake studies that explore potential variations in administration routes, with the objective of enhancing the selectivity of these nanoparticles towards the site of infection. Despite the paucity of studies conducted on the application of these substances, extant data suggest that nanoparticles may emerge as a new type of treatment for fungal infections associated with biofilm formation.

## Figures and Tables

**Table 1:** Nanoparticles active on microorganism’s biofilm divided on principal constituent.

Nanoparticle Type	Coating Agents	Fungal Microorganism Tested	MIC Concentration Range	Reference
**Gold**	β-caryophyllene	*C. albicans*, *C. albicans*-*S. aureus* (RS)	512 μg/mL	[151]
	Phloroglucinol (PG)	*C. albicans*, *C. albicans*-*S. aureus* (RS)	2048 μg/mL	[152]
	Fucoidan	*C. albicans*-*S. aureus* *, *C. albicans*-*S. mutans* (RS)	1024 μg/mL	[153]
	Resveratrol (RSV)	*C. albicans* (RS), *C. albicans* (CI)	2.46 μg/mL	[154]
	*Crinum latifolium*	*C. albicans*, *C. dubliniensis*, *C. krusei*, *C. parapsilosis*, *C. tropicalis* (RS)	250–500 μg/mL	[155]
	Indolicidin	*C. albicans* (RS), *C. albicans*, *C. tropicalis* (CI)	150–200 μg/mL	[156]
	Chytosan–Tyrosol	*C. albicans*, *C. glabrata* (RS)	200–400 μg/mL	[157]
**Silver**	*Erodium glaucophyllum*	*C. albicans* (CI)	50 μg/mL	[158]
	*Encephalartos laurentinus*	*C. albicans* (CI)	8–256 μg/mL	[159]
	Polyvinylpyrrolidone (PVP)	*C. auris* (CI)	0.7–32 μg/mL	[160]
	*Anabaena variabilis*	*C. albicans* (RS)	12.5 μg/mL	[161]
	*Vitis* *vinifera*	*C. albicans* (RS)	20 μg/mL	[162]
	*Phanerochaete chrysosporium*	*C. albicans* (RS)	2 μg/mL	[163]
	*Eucalyptus camaldulensis*	*C. albicans* (CI), *C. albicans* (RS)	2 μg/mL	[164]
	*Salvia officinalis* (* Ag-Ni NPs)	*C. albicans* (RS)	3.12 μg/mL	[165]
	Citrate	*C. albicans*, *C dubliniensis*, *C. krusei*, *C. parapsilosis*, *C. tropicalis*, *L. elongisporus* (RS)	37.5–75 μg/mL	[166]
	Polyethyleneimine (PEI)	*C. albicans*, *C. parapsilosis*, (*Issatchenkia orientalis*) *C. krusei* (RS), *C. albicans*, *C. glabrata*, *C. parapsilosis*, *C. tropicalis* (CI)	0.078–1.25 μg/mL	[167]
	*Penicillium fimorum* metabolites	*C. albicans* (RS)	4 μg/mL	[168]
	*Terminalia catappa*	*C. albicans* (RS)	31.25–250 μg/mL	[169]
	*A. fumigatus*	*A. flavus*, *C. albicans* (RS)	5–11 mg/mL	[170]
	Polyvinylpyrrolidone (PVP)	*C. albicans* (RS), *C. albicans* (CI)	32–128 μg/mL	[171]
	*Aspergillus oryzae* metabolites	*C. albicans*, *C. glabrata*, *C. krusei*, *C. parapsilosis*, *C. tropicalis* (CI)	50–100 μg/mL	[172]
	κ-Carrageenan	*C. albicans*, *C. glabrata* (RS)	50–500 μg/mL	[173]
	none	*C. auris* (CI)	nd	[174]
	none	*A. niger*, *F. oxysporum* (EI)	428 μg/mL	[175]
**Iron Oxide**	*Tamarindus indica*	*C. albicans* (RS)	20.7 μg/mL	[176]
	*Aloe vera*–Cobalt	*C. albicans* (RS)	1000 μg/mL	[177]
	Chitosan	*C. albicans*-*C. glabrata*-*C. tropicalis* (RS)	39–156 μg/mL	[178]
**Zinc Oxide**	Silver nanoparticles	*T. mentagrophytes* (RS)	n.d.	[179]
	*Lactobacillus salivaris*	*C. albicans* (CI)	10–20 μg/mL	[180]
	Lignin	*C. albicans* (RS)	n.d.	[181]
	*Lactobaciluss gasseri*	*C. auris* (CI)	61.9–151 μg/mL	[182]
**Chitosan**	Phloroglucinol	*C. albicans*-*K. pneumoniae*/*S. aureus*/*S. mutans* (RS)	2048 μg/mL	[77]
	Curcumin	*C. albicans*-*S. aureus* (RS)	200–400 μg/mL	[183]
	Olea europaea	*C. albicans* (RS)	n.d.	[184]
	Berberine (BBR)	*C. albicans* (RS)	n.d.	[185]
	Polyanionic sodium triphosphate (TPP)	*C. albicans*, *C. albicans*-*S. aureus* (RS)	n.d.	[186]
	*Apis mellifera* venom	*C. albicans* (RS)	1.56–3.12 μg/mL	[187]
	Salicylhydrazide	*C. albicans* (RS)	3.9–125 μg/mL	[188]
	Carvacrol	*C. albicans*, *C. glabrata*, *C. krusei*, *C. tropicalis* (CI)	24–1560 μg/mL	[189]
**Liposome**	Anidulafungin	*C. albicans* (RS)	1.56–12.50 μg/mL	[124]
	Soy lecithin (SL), Lauric acid (LA) * Soy lecithin (SL), Myristoleic acid (MA) *	*C. albicans*, *C. albicans-S. aureus* (RS)	n.d.	[190]
**Polylactic Acid**	Ketoconazole	*C. albicans*, *C. dubliniensis*, *C. krusei*, *C. parapsilosis*, *C. tropicalis*, *T. rubrum*, *T. mentagrophytes*, *M. gypseum* (RS)	0.007–0.015 μg/mL	[191]
	pterostilbene (PTB), pomace extracts	*C. albicans* (RS)	>16 μg/mL (PTB) 50 μg/mL pomace	[192]
	pterostilbene (PTB), pomace extracts	*A. brasiliensis* (RS)	n.d.	[193]
**Carbon based**	[CD] NH_2_, CO_2_H/NH_2_, CO_2_H	*C. albicans* (RS)	(NH_2_) 397 μg/mL; (CO_2_H/NH_2_, CO_2_H) > 500 μg/mL	[141]
	[CD] Nitrogen, Silver embedded	*C. albicans* (RS)	4–256 μg/mL	[194]
	[GO] none	*C. albicans*-*S. aureus* (RS)	n.d.	[195]
	[GO] Curcumin	*C. parapsilosis* (CI)	n.d.	[196]

* Not coating agents but components of the structure; mixed biofilm (fungal–bacteria or fungal–fungal); CI: clinical isolate; RS: reference strain; EI: environmental isolate; n.d.: not defined; [CD]: carbon dots; [GO]: graphene oxide.

**Table 2:** In vivo studies of nanoparticles tested on fungal biofilm.

**In Vivo Model**	**Nanoparticle Type**	**Study Conducted**	**Reference**
**Murine**	Silver coated with *Polyvinylpyrrolidone* (PVP)	Systemic candidiasis treatment	[171]
	Zinc oxide coated with Lignin	Cytotoxicity of nanoparticles	[181]
	Silver coated with *Erodium glaucophyllum*	Oral candidiasis treatment	[158]
**Larvae** **(*Galleria mellonella*)**	Liposome coated with Anidulafungin	Systemic candidiasis treatment	[124]
	Carbon dots coated with NH_2_, CO_2_H/NH_2_, CO_2_	Systemic candidiasis treatment	[141]
	Polylactic acid coated with pterostilbene (PTB), pomace ex-tracts	*A. brasiliensis* systemic infection treatment and cytotoxicity of nanoparticles	[193]

## Data Availability

No new data were created or analyzed in this study. Data sharing is not applicable to this article.
